# *SlZRT2* Encodes a ZIP Family Zn Transporter With Dual Localization in the Ectomycorrhizal Fungus *Suillus luteus*

**DOI:** 10.3389/fmicb.2019.02251

**Published:** 2019-10-10

**Authors:** Laura Coninx, Nick Smisdom, Annegret Kohler, Natascha Arnauts, Marcel Ameloot, François Rineau, Jan V. Colpaert, Joske Ruytinx

**Affiliations:** ^1^Centre for Environmental Sciences, Environmental Biology, Hasselt University, Diepenbeek, Belgium; ^2^Biomedical Research Institute, Hasselt University, Diepenbeek, Belgium; ^3^Laboratoire d’Excellence ARBRE, Institut National de la Recherche Agronomique, UMR 1136 INRA/Université de Lorraine Interactions Arbres/Microorganismes, Champenoux, France

**Keywords:** Mycorrhiza, *Suillus luteus*, zinc transporter, zinc homeostasis, ZIP

## Abstract

Ectomycorrhizal (ECM) fungi are important root symbionts of trees, as they can have significant effects on the nutrient status of plants. In polluted environments, particular ECM fungi can protect their host tree from Zn toxicity by restricting the transfer of Zn while securing supply of essential nutrients. However, mechanisms and regulation of cellular Zn homeostasis in ECM fungi are largely unknown, and it remains unclear how ECM fungi affect the Zn status of their host plants. This study focuses on the characterization of a ZIP (Zrt/IrtT-like protein) transporter, *SlZRT2*, in the ECM fungus *Suillus luteus*, a common root symbiont of young pine trees. *SlZRT2* is predicted to encode a plasma membrane-located Zn importer. Heterologous expression of *SlZRT2* in yeast mutants with impaired Zn uptake resulted in a minor impact on cellular Zn accumulation and growth. The *SlZRT2* gene product showed a dual localization and was detected at the plasma membrane and perinuclear region. *S. luteus* ZIP-family Zn uptake transporters did not show the potential to induce trehalase activity in yeast and to function as Zn sensors. In response to excess environmental Zn, gene expression analysis demonstrated a rapid but minor and transient decrease in *SlZRT2* transcript level. In ECM root tips, the gene is upregulated. Whether this regulation is due to limited Zn availability at the fungal–plant interface or to developmental processes is unclear. Altogether, our results suggest a function for *SlZRT2* in cellular Zn redistribution from the ER next to a putative role in Zn uptake in *S. luteus.*

## Introduction

Mycorrhizae are omnipresent mutualistic associations between fungi and plant roots. Mycorrhizal fungi provide their host plants with nutrients in exchange for sugar and/or lipids (Martin et al., 2016; Keymer et al., 2017). In addition to the supply of nutrients, host plants may benefit from an improved resistance for organic and inorganic pollutants (Adriaensen et al., 2004; Cabral et al., 2015; Ferrol et al., 2016). Therefore, the use of mycorrhizal plants is considered in phytoremediation applications (Coninx et al., 2017a) and in strategies to improve the nutritional quality of crops (Sharma et al., 2017). As Zn deficiency and Zn toxicity are frequently observed in plants, mycorrhizal fungi with the ability to enhance or reduce Zn transfer to the plant can be crucial for the success of phytoremediation or biofortification applications (Adriaensen et al., 2004; Cavagnaro, 2008; Yang et al., 2015; Miransari, 2017). Zn deficiency is the most widespread and recurrent micronutrient deficiency in pasture and crop plants worldwide (Alloway, 2004), whereas the less prevalent Zn toxicity is generally reported for plants growing in the vicinity of mining or metallurgic plants (Ernst, 1990; Alloway, 2004; Nagajyoti et al., 2010).

Zn deficiency can have severe physiological consequences in plants and mycorrhizal fungi, as Zn is an essential micronutrient that ensures the structural stability and catalytic activity of many proteins. Organisms must maintain adequate intracellular concentrations of Zn (usually between 0.1 and 0.5 mM total cellular Zn), even when extracellular Zn levels are low (Eide, 2006). In order to meet this high demand for Zn, cells primarily rely on integral membrane transport proteins (Gaither and Eide, 2001). Yet, unbound cytoplasmic Zn levels are kept to a minimum in the cell, since free Zn ions can cause harmful effects. Proteins can be damaged or inactivated by the uncontrolled binding of Zn ions to functional groups in these proteins (Gaither and Eide, 2001). In order to avoid Zn toxicity, cells rely on a wide range of Zn homeostasis mechanisms that are strictly regulated. Upon entry into the cell via specialized transporter systems, Zn is either chelated intracellularly by various ligands (e.g., metallothioneins) or sequestered into subcellular compartments by transporter proteins (Becquer et al., 2019). Excess Zn can be removed from the cell via an enhanced efflux (Becquer et al., 2019). Given their crucial role in Zn efflux, uptake, and sequestration, transporter proteins are considered indispensable for the cellular Zn metabolism.

Fungal Zn transporters have mainly been identified in two protein families: the ZIP (Zrt/Irt-like protein) and CDF (Cation Diffusion Facilitator) transporter families (Eide, 2006). These two protein families also include iron (Fe) and manganese (Mn) transporters. Several CDF and ZIP proteins have been demonstrated to transport, to a lesser extent, other divalent metal ions, such as cadmium (Cd), cobalt (Co), and nickel (Ni) (Guerinot, 2000; Montanini et al., 2007). While ZIP transporters are known to transport extracellular or stored Zn into the cytoplasm (Kambe et al., 2006), CDFs decrease cytoplasmic Zn levels by transporting Zn into organelles or out of the cell (Montanini et al., 2007). Several CDFs have been described in ectomycorrhizal (ECM) fungi. In *Hebeloma cylindrosporum*, Zn storage in endoplasmic reticulum (ER)-derived vesicles is mediated by HcZnT1 (Blaudez and Chalot, 2011). Vacuolar Zn storage in *Suillus luteus* (Ruytinx et al., 2017) and in *Russula atropurpurea* (Sacky et al., 2016) is governed by the CDF transporters SlZnT1 and RaCDF1, respectively. Zn export in *R. atropurpurea* is accomplished by the RaCDF2 transporter (Sacky et al., 2016). Two ZIP transporters, RaZIP1 and SlZRT1, which are involved in high-affinity Zn uptake, were described in *R. atropurpurea* (Leonhardt et al., 2018) and *S. luteus* (Coninx et al., 2017b).

Recently, it has been reported that the ZIP transporter ScZRT1, which is one of the two principal plasma membrane-located Zn uptake systems of yeast, has a role in Zn sensing (Schothorst et al., 2017). ScZRT1 governs rapid activation of the PKA (protein kinase A) pathway upon Zn repletion of Zn-deprived yeast cells. This results in a quick exit from the stationary growth phase and a rapid surge in the activity of trehalase, which is a well-established PKA target (Thevelein and de Winde, 1999). ScZRT1 is likely crucial for a swift response to abrupt changes in environmental Zn availability, illustrating the significance of ZIP transporters not only in maintaining the cellular Zn homeostasis but also in regulating the adaptive growth response. In the present study, we aim at characterizing a putative plasma membrane-located ZIP transporter, SlZRT2, in the ECM fungus *S. luteus* and investigate whether *S. luteus* ZIP transporters have the potential to function as Zn sensors. *S. luteus* is an ECM model system and a cosmopolitan pioneer fungus that associates with the roots of young pine trees. The species supports pine seedling establishment (Hayward et al., 2015) and Zn-tolerant suilloid isolates have been demonstrated to protect their host plants from Zn toxicity in Zn-polluted soils (Adriaensen et al., 2004). These features make *S. luteus* an interesting candidate for use in phytostabilization applications (Coninx et al., 2017b). However, a comprehensive understanding of Zn metabolism in *S. luteus* and how the fungus affects the host’s Zn status is crucial for the development of such a strategy. While the ability of ECM fungi to decrease or increase the transfer of Zn to the host plant is well-recognized (Colpaert et al., 2011; Langer et al., 2012; Becquer et al., 2019), little is known of the molecular mechanisms involved.

## Materials and Methods

### *S. luteus* Strains and Culture Conditions

The dikaryotic *S. luteus isolate* UH-Slu-P4 (Colpaert et al., 2004) was used in this study. The fungal isolate was maintained in culture on modified solid Fries medium (Colpaert et al., 2004). Liquid cultures of UH-Slu-P4 were initiated for use in the gene expression assay as described by Nguyen et al. (2017). Cultures were treated to induce Zn deficiency, Zn sufficiency, and mild Zn toxicity (0, 20, 500, or 1000 mM ZnSO_4_.7H_2_O) according to Coninx et al. (2017b). Zn-exposed mycelia (400 mg) were sampled at 1, 2, 4, 8, and 24 h after initiation of Zn treatment, flash frozen with liquid N_2_, and stored at −70°C.

### *SlZnT2* Sequence Analysis

The ZIP transporter *SlZRT2* was previously identified in the genome of *S. luteus* UH-Slu-Lm8-n1 v2.0 (Coninx et al., 2017b). The corresponding amino acid sequence was further investigated *in silico*. Transmembrane domains (TMDs) were predicted by the topology prediction program TMHMM2.0 (Krogh et al., 2001). Amino acid sequence similarities were calculated with Sequence Manipulation Suite version 2 (Stothard, 2000). A subcellular localization prediction was performed with “ProtComp v.9.0. Identifying sub-cellular location (Animals and Fungi)” from Softberry^1^. SlZRT2 and ScZRT2 (Eide, 1996) were aligned with Multiple Alignment using Fast Fourier Transform version 7 (MAFFT) (Katoh and Standley, 2013).

### SlZRT2 Cloning and Heterologous Expression in Yeast

A cDNA library of the sequenced isolate UH-Slu-Lm8-n1 (Kohler et al., 2015) was constructed according to Coninx et al. (2017b). A gene-specific primer pair was developed to amplify the full-length coding sequence of *SlZRT2* (forward primer: 5′ TCAGCACTTCACCACAGGCTTACTATC 3′; reverse primer: 5′ CATCCCCACGAGCGCCAT 3′). A 30-μl PCR reaction was performed according to Coninx et al. (2017b). Reaction specificity and amplicon length were verified by visualization of 5-μl PCR product on a 1.5% agarose gel with GelRed^®^ Nucleic Acid Gel Stain (Biotium, Fremont, CA, United States). The remaining PCR product (25 μl) was processed with the GeneJet PCR purification kit (Thermo Scientific, Waltham, MA, United States). Subsequently, the purified amplicon was cloned into the gateway entry vector pCR8/GW/TOPO (Invitrogen, Carlsbad, CA, United States) and transferred to the destination vectors pAG426GAL-ccdB-EGFP (Alberti et al., 2007) and pYES-DEST52 (Invitrogen, Carlsbad, CA, United States) with the Gateway LR-clonase II Enzyme Mix (Invitrogen, Carlsbad, CA, United States) according to the manufacturer’s instructions.

### Yeast Mutant Phenotype Complementation

The following yeast strains were used for the heterologous expression of *SlZRT2*: CM30 (MATa, ade6, can1-100, his3-11, 15leu2-3, trp1-1, ura3-52), CM34 or Δzrt1Δzrt2 (CM30, zrt1:LEU2, zrt2:HIS3) (MacDiarmid et al., 2000), BY4741 (MAT a; his3Δ1; leu2Δ; met15Δ0; ura3Δ0), Δsmf1 (BY4741; MATa; ura3Δ0; leu2Δ0; his3Δ1; met15Δ0; YOL122c:kanMX4), and Δftr1 (BY4741; MATa; ura3Δ0; leu2Δ0; his3Δ1; met15Δ0; YER145c:kanMX4) (EUROSCARF, Frankfurt, Germany). High-efficiency yeast transformation was performed with the LiAc/PEG method as described by Gietz and Woods (2002). Selection of the transformed yeast cells and phenotypic screening (drop assays) of the yeasts were performed as described by Coninx et al. (2017b). To induce protein production using the GAL1 promoter, induction SD-URA medium (synthetic defined medium with 20 g l^–1^ galactose instead of glucose and without uracil) was used. Yeast assays with Δzrt1Δzrt2 strains were performed in petri dishes with solid SD-URA induction medium supplemented with 1 mM ZnSO_4_.7H_2_O (control) or 0.5 and 2 mM trisodium citrate to restrict Zn availability in the growth medium. SD-URA induction medium supplemented with 100 μM MnSO_4_.H_2_O (control) or 8 and 15 mM egtazic acid (EGTA) was used in the complementation assays with Δsmf1 strains. Assays with Δftr1 strains were performed on SD-URA induction medium supplemented with 100 μM FeCl.6H_2_O (control) or 10 and 25 μM ethylenediaminetetraacetic acid (EDTA). EGTA and EDTA were supplemented to the SD-URA induction medium to limit the availability of Mn and Fe, respectively. All assays were performed in triplicate using independent yeast clones.

### Subcellular Localization of the SlZRT2:EGFP Fusion Protein

Yeast Δzrt1Δzrt2 cells expressing SlZRT2:EGFP (enhanced green fluorescence protein) translational fusion proteins were grown to mid-log phase OD_600_ = 1 on SD-URA induction medium with 100 μM ZnSO_4_.7H_2_O. Staining of the plasma membrane and vacuolar membrane was performed with FM4-64 (Molecular Probes, Invitrogen, Carlsbad, CA, United States) at 0 and 30°C, respectively, as described by Vida and Emr (1995). When applied at 30°C, this lipophilic dye ends up in the vacuolar membrane of living yeast cells through an endocytic pathway. Application of the same dye in yeast cultures kept on ice (0°C) results in plasma membrane staining due to the inhibition of endocytosis by low temperatures (Vida and Emr, 1995). Nuclei were visualized with the cell-permeant nuclear counterstain Hoechst 33342 (Invitrogen, Carlsbad, CA, United States). Hoechst 33342 was added at a final concentration of 10 μg ml^–1^ to an aliquot of yeast culture in liquid SD-URA induction medium with 100 μM ZnSO_4_.7H_2_O. Yeast cells were incubated in the dark at 30°C for 40 min.

Imaging of the FM4-64 vacuolar and plasma membrane staining in EGFP-expressing yeast cells was performed with a Zeiss LSM 880 laser scanning confocal microscope (Carl Zeiss, Jena, Germany) mounted on an inverted microscope Axio observer (Carl Zeiss, Jena, Germany) using a Zeiss 63 × NA1.2 water immersion C-Apochromat objective. EGFP and FM4-64 were simultaneously excited using, respectively, the 488-nm laser line of an argon-ion laser and the 633-nm laser line of a helium–neon laser. An MBS 488/543/633 beam splitter was used to separate fluorescence emission light from this excitation light. The resulting emission light of EGFP and FM4-64 was detected using, respectively, the wavelength range of 490 to 570 nm of the spectral GaAsP detector and the wavelength range of 637 to 758 nm detected by a PMT. An image resolution of 512 by 512 pixels was used, with a pixel dwell time of 8.19 μs and a pixel size of 70 nm.

Hoechst 33342-stained EGFP-expressing yeast cells were analyzed with an Elyra PS.1 (Carl Zeiss, Jena, Germany), using a Zeiss 100 × NA1.46 oil immersion alpha Plan-Apochromat objective. A sequential recoding of two channels was performed. Hoechst 33342 was excited using a 405-nm laser line and emission was collected through a BP 420–490 nm band-pass filter. EGFP was excited using a 488-nm laser line and emission was collected through a BP 495–575 nm band-pass filter. The image was recorded using an EM-CCD camera (Andor) with a resolution of 512 by 512 pixels, a pixel size of 100 nm, and an exposure time of 300 ms. Image processing was carried out with ZEN 2.3 (blue edition) Service Pack 1 Software (Carl Zeiss, Jena).

### Zn Content Analysis of Transformed Yeasts

Transformed yeast cells were grown in liquid induction medium with 100 μM ZnSO_4_.7H_2_O at 30°C to mid log phase (OD_600_ ± 1.5). After dilution to OD_600_ = 1, 1 ml of yeast suspension was added to 20 ml of liquid induction medium with 100 μM ZnSO_4_.7H_2_O. Five independent yeast clones of each transformed yeast strain were tested. Cultures were grown for 24 h at 30°C. Yeast cells were collected by centrifugation and washed three times with 20 mM PbNO_3_ and milli-Q water. Collected cells were resuspended, lyophilized, and acid digested according to Coninx et al. (2017b). Zn content was analyzed with inductively coupled plasma optical emission spectrometry (ICP-OES).

### Trehalase Activity in Transformed Yeasts

Transformed yeast cells were grown in liquid induction medium supplemented with 500 μM trisodium citrate at 30°C until culture saturation. Saturated yeast suspension (0.5 ml) was re-inoculated in fresh induction medium with 500 μM trisodium citrate. Zn-deprived cells were grown for 2 h in Zn limitation medium containing 10 mM trisodium citrate and 1 mM EDTA and treated with 5 mM ZnCl_2_. Culture aliquots containing 75 mg (wet weight) cells were taken at −5, 0, 1, and 4 min after treatment with Zn. Yeast cells were resuspended in 45 ml of ice-cold water and harvested by centrifugation. Crude protein extracts were prepared according to Pernambuco et al. (1996).

Crude extracts were dialyzed as described by Van Houtte and Van Dijck (2013) with the Pierce^TM^ 96-well Microdialysis Plate (10K MWCO) (Thermo Fisher, Waltham, MA, United States). Trehalase activity assay and data analysis were performed according to Van Houtte and Van Dijck (2013).

### Gene Expression Analysis in *S. luteus*

The effect of the extracellular Zn concentration on the expression of *SlZRT2* was analyzed in the isolate UH-Slu-P4 via reverse transcription quantitative polymerase chain reaction (RT-qPCR). The experimental setup and data analysis were performed as described by Coninx et al. (2017b). For the amplification of *SlZRT2*, a gene-specific primer pair was designed using Primer3web version 4.1.0 (Rozen and Skaletsky, 2000) (forward primer: 5′ TTCTACGCTCTCACTCGAAG 3′; reverse primer: 5′ CGGTGAACTGTATGACTGGA 3′, primer efficiency = 96.9%). Expression data were normalized with the reference genes TUB1, ACT1, AM085296, AM085296, and GR97562 (Ruytinx et al., 2016). Reference gene expression stability was confirmed within the current experimental setup using GeNorm (Vandesompele et al., 2002). The geometric mean of the relative expression levels of the reference genes was calculated and applied as a normalization factor. Gene expression data were expressed relative to the sample with the highest expression level via the formula 2^–Δ^ ^Ct^.

Mean values of the biological replicates (*n* = 4) were calculated, rescaled to the 20 μM Zn (control) condition within each time point, and log2 transformed. To assess differences in *SlZRT2* expression levels, a two-way analysis of variance (ANOVA) followed by a Tukey’s HSD test was performed in “R” version 3.2.2 (R Core Team, 2012).

The transcript profiles of *SlZRT1* and *SlZRT2* were analyzed in free living mycelium (FML) UH-Slu-Lm8-n1 (Kohler et al., 2015) and in symbiotic *S. luteus–Pinus sylvestris* ECM root tips. Expression data were obtained from the GSE63947 expression data set, which is published (Kohler et al., 2015) and can be accessed via the Gene Expression Omnibus at the NCBI website (National Center for Biotechnology Information^2^). Significant differences in *SlZRT1* and *SlZRT2* expression were assessed with the Welch Two Sample *t* test using “R” version 3.2.2 (R Core Team, 2012).

### Zn Accumulation in *S. luteus*

The Zn content of the *S. luteus* isolate UH-Slu-P4 was analyzed at multiple time points following Zn exposure. The experimental setup was identical to the one used in the RT-qPCR assay. At each exposure time point, four aliquots of mycelium (± 50 mg wet weight) were collected in 2-ml Eppendorf tubes and washed three times with 20 mM PbNO_3_ and milli-Q water. Mycelial samples were lyophilized and acid digested, and Zn content was determined by ICP-OES.

## Results

### SlZRT2 Sequence Analysis

SlZRT1 and SlZRT2, two homologs of the yeast ZIP transporters ScZRT1 and ScZRT2, were recently identified in the genome of *S. luteus.* SlZRT1 functions as a high-affinity plasma membrane-located Zn transporter (Coninx et al., 2017b).

**FIGURE 1:**
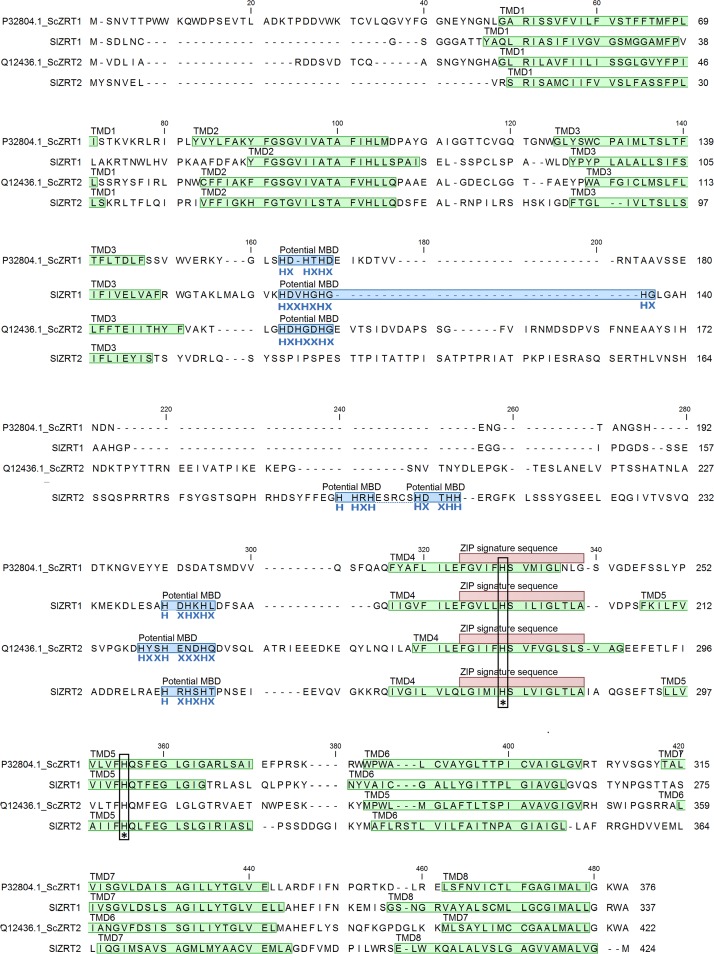
SlZRT1, SlZRT2, ScZRT1, and ScZRT2 protein sequence. Transmembrane domains (TMDs) as predicted by TMHMM2.0, are indicated in green, putative histidine-rich metal binding domains (MBDs) in blue, and the ZIP signature sequence (Eng et al., 1998) in red. Two among ZIP proteins conserved histidines (located in TMD4 and TMD5) are indicated with an asterisk.

*SlZRT2* is predicted to have a 1678-bp open reading frame consisting of eight exons. These eight exons encode a 425-amino-acid polypeptide (jgi prot ID 720881), which demonstrates several typical ZIP transporter features (Figure 1). SlZRT2 is predicted to localize to the plasma membrane and to have eight TMDs and a long variable cytoplasmic loop between TMD3 and TMD4. The variable cytoplasmic loop contains several histidine-rich motifs (HHXH, CXHXXHH, HXHXHX) that could function as binding site(s) for Zn and/or other metals, so-called metal binding domains (MBDs). ZIP signature sequence (Eng et al., 1998) and typically conserved histidines were identified (Figure 1). First two histidine-rich motifs are separated by only five amino acids and might be considered as one. As the previously characterized SlZRT1, SlZRT2 shows a high degree of sequence similarity with the yeast ZIP transporters ScZRT1 and ScZRT2. Though considerable sequence variation is observed within the cytoplasmic loop between TMD3 and TMD4. In SlZRT2, the first histidine-rich motif is preceded by a long amino acid stretch and is somehow shifted toward the second histidine-rich motif when compared with ScZRT2.

### Heterologous Expression of SlZRT2 in Yeast

**FIGURE 2:**
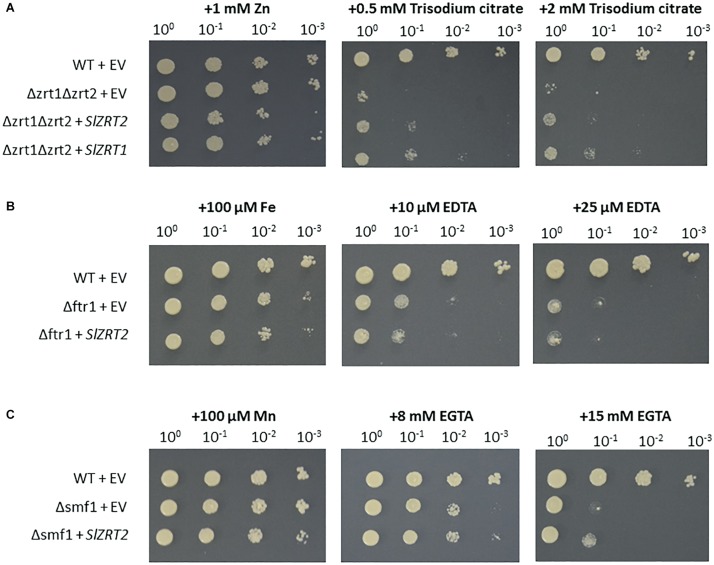
Functional complementation assays of Zn, Fe, and Mn uptake-deficient yeast strains Δzrt1Δzrt2 **(A)**, Δftr1 **(B)**, and Δsmf1 **(C)**. Cultures of wild-type (WT) and mutant yeast cells (OD_600_ = 1) were 10-fold serial diluted (10^0^, 10^–1^, 10^–2^, and 10^–3^) and spotted on control (first column) or selection SD medium (second and third column). Control medium was supplemented with Zn, Fe, or Mn and selection medium with different concentrations of citrate, EDTA, or EGTA. WT cells were transformed with the empty vector (EV, pYES-DEST52; Invitrogen). Yeast mutants were transformed with the EV or the vector containing *SlZRT2* or previously characterized *SlZRT1*. Pictures were taken after 3 days of growth and experiments were carried out for three independent clones.

Heterologous expression with the empty vector (EV) did not result in complementation of any metal uptake-deficient phenotype (Figures 2A–C and Supplementary Figures S1A,B), whereas growth of the Zn uptake-deficient yeast mutant Δzrt1Δzrt2 was partially restored by heterologous expression of *SlZRT2* (Figure 2A and Supplementary Figures S1A,B). When compared to *SlZRT1*, *SlZRT2* is less able to complement the Zn-deficient phenotype of Δzrt1Δzrt2. *SlZRT2* is not able to complement the Fe uptake-deficient phenotype of Δftr1 (Figure 2B). However, growth of the Mn uptake-deficient yeast mutant Δsmf1 is marginally restored (Figure 2C).

**FIGURE 3:**
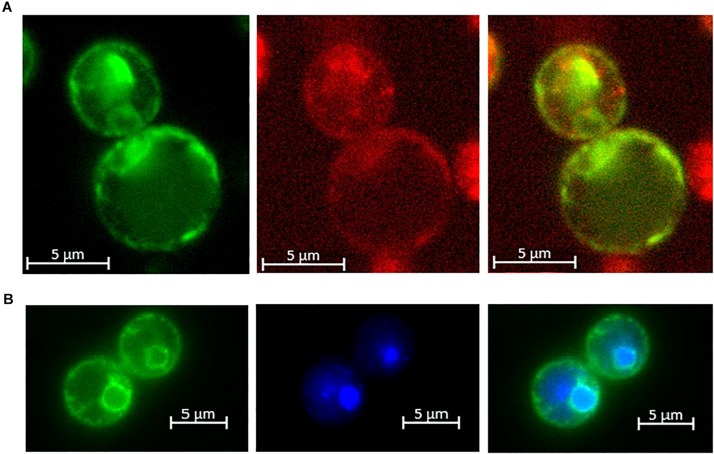
Localization of the SlZRT2:EGFP fusion protein in yeast. **(A)** FM4-64 plasma membrane staining on ice to avoid endocytosis of the dye and **(B)** Hoechst 33324 nuclear staining. From left to right, pictures visualize the EGFP (green) fusion protein (left), FM4-64 (red) or Hoechst (blue) staining (middle), and the merged image (right).

Yeast cells expressing the SlZRT2:EGFP fusion protein (Figures 3A,B and Supplementary Figure S2A) displayed GFP fluorescence at two distinct subcellular locations. An outer green fluorescent ring co-localizes with FM4-64 plasma membrane staining at 30°C (Figure 3A) and GFP fluorescence in the perinuclear region surrounding the Hoechst33342 nucleic acid stain (Figure 3B). No co-localization of EGFP fluorescence with FM4-64 vacuolar staining (0°C) was observed (Supplementary Figure S2A). EGFP fluorescence of EV transformed yeast cells was solely detected in the cytoplasm (Supplementary Figures S2B,C).

### Zn Content Analysis of SlZRT2 Transformed Yeast Cells

**FIGURE 4:**
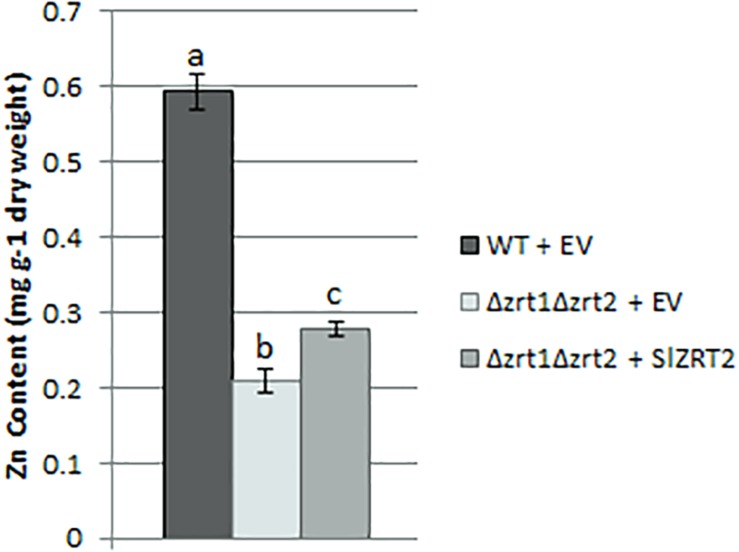
Zn content of WT and Δzrt1Δzrt2 transformed yeast cells transformed with the EV (pYES-DEST52; Invitrogen) or the vector containing *SlZRT2*. Data are the average ± standard error (SE) of five replicates; significant differences (*p* < 0.05) are indicated by different letters.

Yeast cultures were grown in standard growth medium supplemented with 100 μM Zn to ensure adequate growth of all yeast mutants. Wild-type (WT) cells transformed with an empty vector (EV) contain significantly more Zn than Δzrt1Δzrt2 mutant cells transformed with the same EV (Figure 4). Δzrt1Δzrt2 mutants transformed with *SlZRT2* have a significantly higher Zn content than the Δzrt1Δzrt2 transformations with the EV. Yet, the Zn content of SlZRT2 transformed mutant cells is still significantly lower than the WT transformations. Similar differences in Zn content were observed in yeast strains transformed with the SlZRT2:EGFP fusion protein (Supplementary Figure S3). No differences in Mn or Fe content were observed in yeast strains with either *SlZRT2* or *SlZRT2:EGFP* heterologous expression (Supplementary Figures S4A,B).

### Trehalase Activity in Zn-Depleted Yeast Cells Re-supplemented With Zn

**FIGURE 5:**
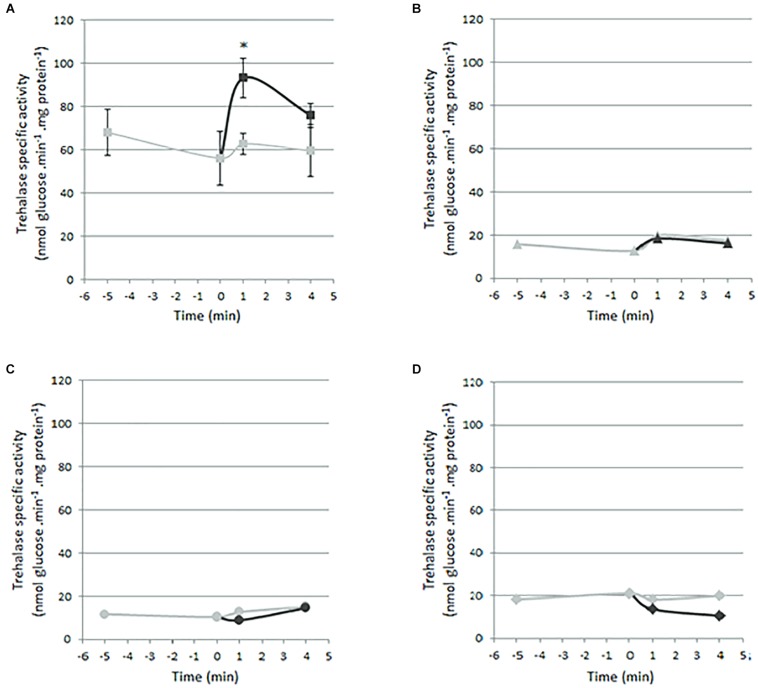
Trehalase activity in Zn-depleted yeast cells after the re-supplementation of Zn. Trehalase activity was assessed in yeast cells maintained on Zn starvation medium (in gray; negative control) and after the addition of 5 mM ZnCl_2_ (in black). **(A)** WT cells transformed with the EV (pYES-DEST52; Invitrogen). Data are the mean ± SE of three biological replicates. Cells were cultured for 2 days in SD medium supplemented with 10 mM citrate and 1 mM EDTA to trigger Zn starvation. **(B,C)** Trehalase activity of Δzrt1Δzrt2 yeast mutant cells. The trehalase assay with Δzrt1Δzrt2 cells was performed three times, each time under a different Zn starvation regime. Representative results from one starvation regime are shown. Cells were cultured for 2 h in SD medium supplemented with 10 mM citrate and 1 mM EDTA to trigger Zn starvation. Δzrt1Δzrt2 cells were transformed with the EV (**B**; pYES-DEST52; Invitrogen), SlZRT1 **(C)**, or SlZRT2 **(D)**. (Results from the two other Zn starvation regimes are shown in Supplementary Figure S5.)

Trehalase activity was assessed in Zn-depleted WT and Δzrt1Δzrt2 cells upon Zn repletion. WT cells were transformed with the EV (Figure 5A) and Δzrt1Δzrt2 yeast cells with the EV (Figure 5B and Supplementary Figures S5A,B), *SlZRT1* (Figure 5C and Supplementary Figures S5A,B), or *SlZRT2* (Figure 5D and Supplementary Figures S5A,B).

Rapid Zn-induced signaling to the PKA pathway via an increase in trehalase activity was only observed in WT yeast cells. Re-addition of Zn to the Zn-deprived WT cells resulted in a significant 1.5-fold increase in trehalase activity after 1 min (*p* = 0.0414, unpaired *t* test) (Figure 5A). No changes in trehalase activity were observed for Δzrt1Δzrt2 cells transformed with the EV, SlZRT1, or SlZRT2.

### Effect of Extracellular Zn on SlZRT2 Expression in *S. luteus*

**FIGURE 6:**
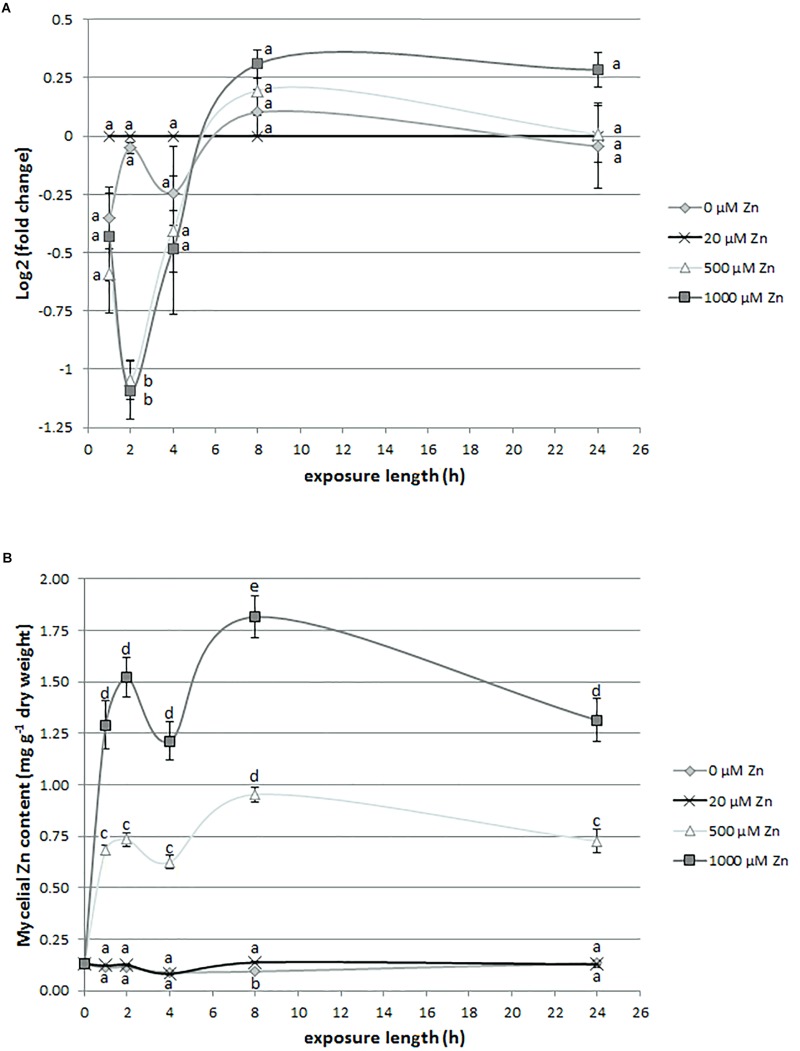
*SlZRT2* expression **(A)** and Zn content **(B)** in *S. luteus* mycelium after 0-, 1-, 2-, 4-, 8-, and 24-h exposure to different concentrations of Zn. Significant differences within each time point are indicated by different letters (*p* < 0.05). **(A)** *SlZRT2* expression data are the average ± SE of three biological replicates and expressed relative in log2(fold change) to the control condition (20 μM Zn) within each time point. **(B)** Mycelial Zn content data are the average ± standard error (SE) of four biological replicates.

*SlZRT2* gene expression was analyzed after short exposure times (1, 2, 4, 8, and 24 h) to different Zn concentrations [0, 20 (control), 500, and 1000 μM]. Figure 6A illustrates that *SlZRT2* mRNA levels are rapidly affected by changes in external Zn concentration. A significant downregulation is observed after 2-h exposure to mildly toxic Zn concentrations (500 and 1000 μM). Despite the difference in Zn concentration, exposure to 500 and 1000 μm Zn resulted in a similar *SlZRT2* expression pattern. Moreover, when external Zn is absent, the *SlZRT2* expression level is also observed to fluctuate in the initial time points (1, 2, and 4 h). At the later time points (8 and 24 h), *SlZRT2* mRNA levels of all the Zn treatments converge and no differences in gene expression are observed.

### Zn Accumulation in *S. luteus* Exposed to Different Concentrations of Zn

Internal mycelial Zn concentrations were measured 1, 2, 4, 8, and 24 h following Zn treatment [0, 20 (control), 500, and 1000 μM] to observe the Zn accumulation pattern in *S. luteus*. While no difference in the Zn accumulation pattern of the 20 μM (control) and 0 μM Zn treatment was observed, treatment with 500 and 1000 μM Zn resulted in a significantly higher Zn uptake (Figure 6B). Although more Zn is accumulated in the 1000 μM treatment, compared to the 500 μM treatment, both Zn exposures have a similar accumulation pattern, with a drop in Zn content at 4 h. This drop occurs shortly after the downregulation in *SlZRT2* expression.

### SlZRT1 and SlZRT2 Transcript Levels in Free-Living *S. luteus* Mycelium and Mycorrhizal Root Tips

To assess whether *SlZRT1* and *SlZRT2* are differentially expressed in *S. luteus* FLM and ECM root tips, RNA-Seq data of these morphological structures were analyzed. Indeed, transcript levels of both ZIP genes are significantly higher in the ECM root tips of the *S. luteus–P. sylvestris* association, when compared to the *in vitro* FLM (Table 1; *p* values <0.05). A sevenfold increase in the expression of *SlZRT1* was observed in ECM root tips. This change in gene activity was less pronounced for *SlZRT2*, for which the transcript levels were 2.3 times higher in the ECM root tips.

**TABLE 1:** *SlZRT1* and *SlZRT2* transcript levels in *S. luteus*–*P. sylvestris* mycorrhizal root tips (ECM) and *S. luteus* free-living mycelium (FLM).

	**ECM (rpkm)**	**FLM (rpkm)**	**Ratio ECM/FLM**
***SlZRT1***	403.7 ± 10.4	57.0 ± 1.4	7.1
***SlZRT2***	31.4 ± 0.2	13.5 ± 0.8	2.3

*RNA-Seq data are the average ± standard error (SE) of two biological replicates and expressed in reads per kilobase million (rpkm).*

## Discussion

Zn is estimated to interact with ∼9% of the eukaryotic proteome to support their structure or catalytic function (Andreini et al., 2006). A tightly controlled cytoplasmic Zn concentration is essential to supply these proteins and overcome malfunction through deficiency or excess. Main gateways for Zn to the cytoplasm are ZIP transporters (Guerinot, 2000). Here, we report on the characterization of a second ZIP transporter, SlZRT2, in the ECM fungus *S. luteus*. SlZRT2 has a high sequence similarity with the previously characterized plasma membrane localized Zn importer, SlZRT1, of the same species (Coninx et al., 2017b) and both high- and low-affinity Zn importers ScZRT1 and ScZRT2 of budding yeast. Nevertheless, considerable sequence variation was observed between TMD3 and TMD4, including two histidine-rich domains. First, the SlZRT2 histidine-rich domain differs in size and position when compared to its homologs. Histidine-rich domains are potential metal binding domains and are assumed to determine substrate specificity and affinity of transporters (Guerinot, 2000). Site-directed mutations of the histidines in the histidine-rich domains of several yeast and human ZIP transporters resulted in a reduced or disrupted Zn uptake capacity (Gitan et al., 2003; Milon et al., 2006; Mao et al., 2007). The variations between the histidine-rich domains of SlZRT2 and ScZRT2 could therefore reflect an altered affinity for Zn or a modified metal specificity.

Heterologous expression of *SlZRT2* in yeast identified Zn as primary substrate and indicated a minor role for SlZRT2 in Zn uptake. SlZRT2 restored the Zn uptake-deficient phenotype of the Δzrt1Δzrt2 yeast mutant only partly and performed significantly less than the previously characterized SlZRT1 considering Zn uptake. Cellular Zn concentrations in SlZRT2 overexpressing Δzrt1Δzrt2 yeast clones were only slightly higher than those in EV transformed Δzrt1Δzrt2 clones (Figure 4). Under the same conditions, SlZRT1 was shown to restore cellular Zn concentrations to the WT level (Coninx et al., 2017b). As mentioned previously, in budding yeast, Zn uptake is governed by two ZIP family transporters, ScZRT1 and ScZRT2. ScZRT1 functions as a high-affinity uptake system in severe Zn limiting conditions (Zhao and Eide, 1996a). Under mild Zn deficiency, ScZRT2 mediates Zn uptake (Zhao and Eide, 1996b). This low-affinity transporter is repressed in low zinc and induced upon re-supplementation (Bird et al., 2004). A similar regulation of Zn uptake might operate in *S. luteus*, using SlZRT1 and SlZRT2 as high- and low-affinity Zn importers, respectively. Next to Zn import, SlZRT2 might contribute at Mn uptake as shown by a reduced sensitivity toward Mn deficiency of SlZRT2 overexpressing Δsmf1 yeast mutants (Figure 2A and Supplementary Figures S1A,B). Different secondary substrates, including Mn, Fe, Cu, and Cd, were suggested for many ZIP proteins (Guerinot, 2000).

The SlZRT2:EGFP fusion protein was observed at two subcellular locations in yeast: the perinuclear region and the plasma membrane (Figures 3A,B and Supplementary Figure S2A). The EGFP signal observed at the perinuclear region likely corresponds to the ER membrane and also the putative plasma membrane localized signal might be mainly due to ER localization. In yeast, the ER is composed of perinuclear ER, which surrounds the nucleus, and of peripheral ER, which is juxtaposed to the plasma membrane (Preuss et al., 1991; Prinz et al., 2000). An ER localization of fungal ZIP family Zn importers was observed previously as an artifact of GFP labeling for ScZRT1 of *Saccharomyces cerevisiae* in particular conditions (Schothorst et al., 2017) or of overexpression for RaZIP1 of *R. atropurpurea* (Leonhardt et al., 2018). We do not expect ER localization of SlZRT2:EGFP to be due to artifacts. In contrast to ScZRT1 and RaZIP1, which are both able to restore the Zn-deficient phenotype of Δzrt1Δzrt2 yeast mutants to a large extent (Zhao and Eide, 1996a; Leonhardt et al., 2018), SlZRT2 makes only a minor contribution to Zn uptake when overexpressed in the Δzrt1Δzrt2 mutant (Figures 2, 4). These results for SlZRT2 were independent of the presence/absence of the EGFP tag (Supplementary Figures S1A, S3). A primary function of SlZRT2 in Zn redistribution from the ER and secondary function as low-affinity Zn uptake mechanism is likely.

Next to their transport function, some plasma membrane-localized transporters function as nutrient sensors (Steyfkens et al., 2018). ScZRT1 of budding yeast is such a protein with dual Zn transport and receptor function. This high-affinity Zn importer activates the PKA pathway and induces downstream trehalase activity upon Zn repletion after a prolonged period of starvation (Schothorst et al., 2017). As the previously characterized SlZRT1, SlZRT2 was not able to induce trehalase activity in yeast (Figures 5B–D and Supplementary Figures S5A,B) and thus translate environmental Zn availability to an adaptive growth response. A currently unknown mechanism, independent from Zn membrane importers, might connect Zn availability to an adaptive growth response in *S. luteus*.

Zn uptake in yeast and many other organisms is controlled at the transcriptional level by Zn availability (Jung, 2015; Wilson and Bird, 2016). In *S. luteus*, transcripts of the high-affinity Zn uptake transporter SlZRT1 swiftly accumulate upon Zn starvation, normalize after a few hours, and are more abundant again after 24 h. In conditions of Zn excess, transcription is significantly downregulated within 2 h and remains low over time (Coninx et al., 2017b). Cellular Zn accumulation pattern in *S. luteus* (Figure 6B) follows the *SlZRT1* transcription level. Zn was also observed to regulate *SlZRT2* transcript levels (Figure 6A). However, the impact of external Zn concentration on transcription level was low and only significant at the early time points upon growth in excess Zn. These gene expression results in *S. luteus* suggest a major function for SlZRT2 in cellular Zn redistribution rather than Zn uptake from the environment, as was also indicated by the heterologous expression experiments in yeast.

In ECM root tips, *SlZRT2* transcript levels are 2.4-fold higher when compared to those in free-living mycelium. This might indicate a need for redistribution of ER-stored Zn pool and a low cytosolic Zn state. Indeed, also high-affinity Zn importer SlZRT1 is highly regulated in ECM root tips with a sevenfold increase in transcript levels. It is unclear (1) whether this high demand for Zn is induced by a low availability of Zn at the plant–fungal interface and (2) if both symbiotic partners are competing for this important micronutrient. Similar increases in transcript level (8×) were noted for the putative ZIP-family Zn importer of the arbuscular mycorrhizal fungus *Rhizophagus irregularis* when comparing intraradical (i.e., symbiotic) and extraradical mycelium (Tamayo et al., 2014). However, increases in transcript level of nutrient uptake transporters in ECM root tips are not restricted to Zn-transporting ZIP-family proteins but were previously observed for the nitrate/nitrite transporter TbNrt2 of *Tuber borchii* (Montanini et al., 2006) and the phosphate transporter HcPT2 of *H. cylindrosporum* (Becquer et al., 2019). Interestingly, the phosphate uptake transporter HcPT2 of *H. cylindrosporum* is indispensable for phosphate release toward the host plant (Becquer et al., 2019). This induces the question whether gene expression of nutrient uptake transporters at the symbiotic interface is regulated by substrate availability or other developmentally related cues and whether they might alter function due to unknown (post-translational) regulatory mechanisms. Rapid mobilization of ER-stored Zn was observed in the fungus *Candida albicans* in response to glucose availability (Kjellerup et al., 2018). A response of ER-localized SlZRT2 to glucose or other plant-derived cues and subsequent Zn release might be part of developmental signaling pathways in *S. luteus*. Zn released from the ER is known to function as a secondary messenger, transducing external signals to result in an adaptive response in animal cells (Yamasaki et al., 2007).

## Conclusion

In conclusion, we characterized a second ZIP transporter, SlZRT2, in *S. luteus*. SlZRT2 localizes to the ER and plasma membrane and likely mediates Zn redistribution in response to Zn availability and developmental stage. A small contribution of SlZRT2 to Zn uptake as a low-affinity plasma membrane-localized transporter is expected. It is unclear whether a putative cytoplasmic Zn release by SlZRT2 in ECM root tips is a response to low Zn availability at the plant–fungal interface or functions as a secondary signal coordinating developmental growth responses. Further investigations of Zn mobilization and involved transporters, including their regulation mechanisms, are necessary to understand how mycorrhizal fungi contribute at an improved Zn status of plants.

## DATA AVAILABILITY STATEMENT

Publicly available datasets were analyzed in this study. This data can be found here: http://www.ncbi.nlm.nih.gov/geo/.

## Author Contributions

LC and JR conceived and designed the experiments. LC, NS, NA, and FR performed the experiments. LC, AK, JC, and JR analyzed and interpreted the data. LC drafted the manuscript. JR edited preliminary versions. All authors reviewed the manuscript and approved the final version.

## Conflict of Interest

The authors declare that the research was conducted in the absence of any commercial or financial relationships that could be construed as a potential conflict of interest.

## Footnotes

1 www.softberry.com

2 http://www.ncbi.nlm.nih.gov/geo/

## Supplementary Material

FIGURE S1 Functional complementation assays of the Zn uptake-deficient yeast strain Δzrt1Δzrt2. Cultures of WT and mutant yeast cells (OD_600_ = 1) were 10-fold serial diluted (10^0^, 10^–1^, 10^–2^, and 10^–3^) and spotted on control (first column) or selection SD medium (second and third columns). Control medium was supplemented with Zn and selection medium with EDTA or citrate, since both substances are known to limit Zn availability in the medium. The supplement concentrations are indicated above the pictures. Pictures were taken after 3 days of growth and experiments were carried out for three independent clones. **(A)** WT cells were transformed with the EV (pAG306GAL-ccdB-EGFP; Alberti et al., 2007). Yeast mutants were transformed with the EV or the vector containing *SlZRT2:EGFP* or *SlZRT1:EGFP*. **(B)** Δzrt1Δzrt2 complementation assay according to Coninx et al. (2017b) with EDTA supplementation in the growth medium. Culture conditions were identical to the ones used in the complementation assay of *SlZRT1* (Coninx et al., 2017b). WT cells were transformed with the EV (pYES-DEST52; Invitrogen). Yeast mutants were transformed with the EV or the vector containing *SlZRT2*.

FIGURE S2 Fluorescence of Δzrt1Δzrt2 yeast cells expressing SlZRT2:EGFP **(A)** or an EV control (**B,C**; pAG306GAL-ccdB-EGFP; Alberti et al., 2007). Cells were visualized for EGFP (left), the counterstaining (middle), or merged images (right). **(A)** FM4-64 vacuolar staining at 30°C to allow endocytosis of the dye by Δzrt1Δzrt2 cells expressing SlZRT2:EGFP. **(B)** FM4-64 vacuolar staining (30°C) and **(C)** Hoechst 33324 nuclear staining of EV transformed Δzrt1Δzrt2 cells.

FIGURE S3 Zn content of WT and Δzrt1Δzrt2 transformed yeast cells transformed with the EV (pAG306GAL-ccdB-EGFP; Alberti et al., 2007) or the vector containing *SlZRT2*. Data are the average ± standard error (SE) of five biological replicates; significant differences are indicated by different letters (*p* < 0.05). For a borderline significant difference, the letter is placed within brackets (*p* ≤ 0.07).

FIGURE S4 Mn **(A)** and Fe **(B)** content of WT and Δzrt1Δzrt2 transformed yeast cells transformed with the EV or the vector containing *SlZRT2*. Yeast cells were transformed with the vector pYES-DEST52 (Invitrogen, left) or with pAG306GAL-ccdB-EGFP (Alberti et al., 2007; right). Data are the average ± standard error (SE) of five replicates; significant differences (*p* < 0.05) are indicated by different letters.

FIGURE S5 Trehalase activity in Zn-depleted Δzrt1Δzrt2 cells after the re-addition of Zn. Δzrt1Δzrt2 cells were transformed with the EV (triangles, pYES-DEST52; Invitrogen), *SlZRT1* (circles), or *SlZRT2* (diamonds). All yeast cultures were grown for 2 days on SD medium with 0.5 mM citrate to induce Zn starvation. Trehalase activity was assessed in yeast cells maintained on Zn starvation medium (in gray; negative control) and after the addition of 1 mM ZnCl_2_ (in black) **(A)** 4.5 h prior to the experiment, yeast cultures were transferred to fresh SD medium with 0.5 mM citrate and 200 μM EDTA. **(B)** 4 h prior to the experiment, yeast cultures were transferred to fresh SD medium with 10 mM citrate and 1 mM EDTA.
