# Supplementary figures and images for: SlZRT2 Encodes a ZIP Family Zn Transporter With Dual Localization in the Ectomycorrhizal Fungus Suillus luteus
Source: Front Microbiol. 2019 Oct 10;10:2251. doi: 10.3389/fmicb.2019.02251 (PMC6797856; doi:10.3389/fmicb.2019.02251)

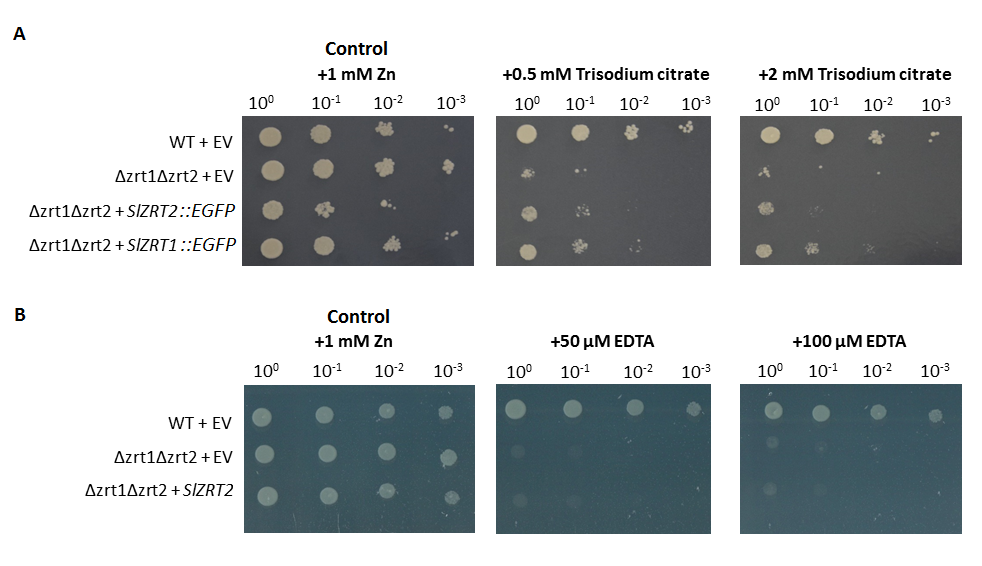

Supplement: FIGURE S1 — Functional complementation assays of the Zn uptake-deficient yeast strain Δzrt1Δzrt2. Cultures of WT and mutant yeast cells (OD600 = 1) were 10-fold serial diluted (100, 10–1, 10–2, and 10–3) and spotted on control (first column) or selection SD medium (second and third columns). Control medium was supplemented with Zn and selection medium with EDTA or citrate, since both substances are known to limit Zn availability in the medium. The supplement concentrations are indicated above the pictures. Pictures were taken after 3 days of growth and experiments were carried out for three independent clones. (A) WT cells were transformed with the EV (pAG306GAL-ccdB-EGFP; Alberti et al., 2007). Yeast mutants were transformed with the EV or the vector containing SlZRT2:EGFP or SlZRT1:EGFP. (B) Δzrt1Δzrt2 complementation assay according to Coninx et al. (2017b) with EDTA supplementation in the growth medium. Culture conditions were identical to the ones used in the complementation assay of SlZRT1 (Coninx et al., 2017b). WT cells were transformed with the EV (pYES-DEST52; Invitrogen). Yeast mutants were transformed with the EV or the vector containing SlZRT2. [file Image_1.TIF]

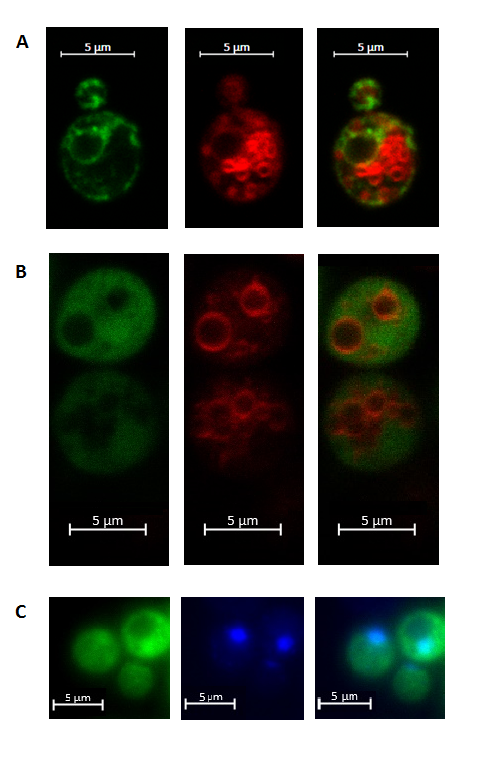

Supplement: FIGURE S2 — Fluorescence of Δzrt1Δzrt2 yeast cells expressing SlZRT2:EGFP (A) or an EV control (B,C; pAG306GAL-ccdB-EGFP; Alberti et al., 2007). Cells were visualized for EGFP (left), the counterstaining (middle), or merged images (right). (A) FM4-64 vacuolar staining at 30°C to allow endocytosis of the dye by Δzrt1Δzrt2 cells expressing SlZRT2:EGFP. (B) FM4-64 vacuolar staining (30°C) and (C) Hoechst 33324 nuclear staining of EV transformed Δzrt1Δzrt2 cells. [file Image_2.TIF]

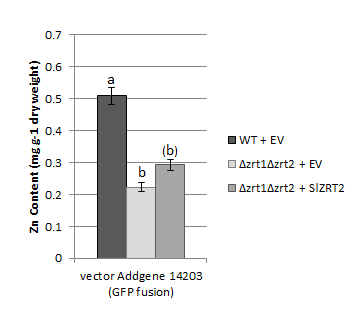

Supplement: FIGURE S3 — Zn content of WT and Δzrt1Δzrt2 transformed yeast cells transformed with the EV (pAG306GAL-ccdB-EGFP; Alberti et al., 2007) or the vector containing SlZRT2. Data are the average ± standard error (SE) of five biological replicates; significant differences are indicated by different letters (p < 0.05). For a borderline significant difference, the letter is placed within brackets (p ≤ 0.07). [file Image_3.TIF]

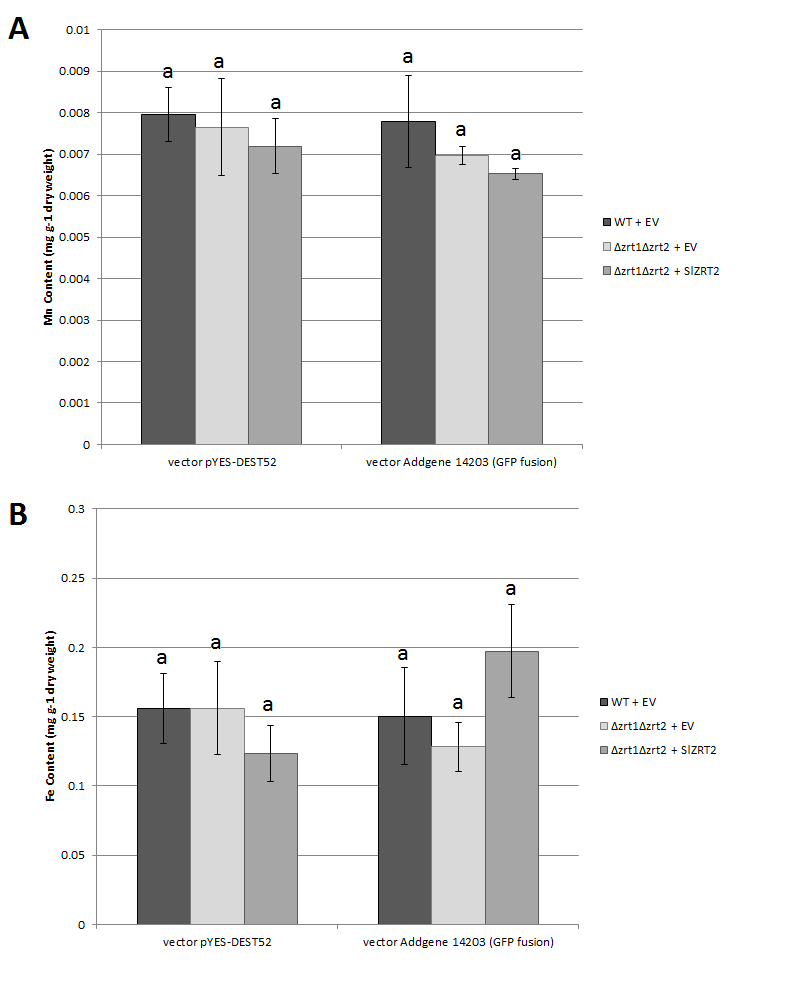

Supplement: FIGURE S4 — Mn (A) and Fe (B) content of WT and Δzrt1Δzrt2 transformed yeast cells transformed with the EV or the vector containing SlZRT2. Yeast cells were transformed with the vector pYES-DEST52 (Invitrogen, left) or with pAG306GAL-ccdB-EGFP (Alberti et al., 2007; right). Data are the average ± standard error (SE) of five replicates; significant differences (p < 0.05) are indicated by different letters. [file Image_4.TIF]

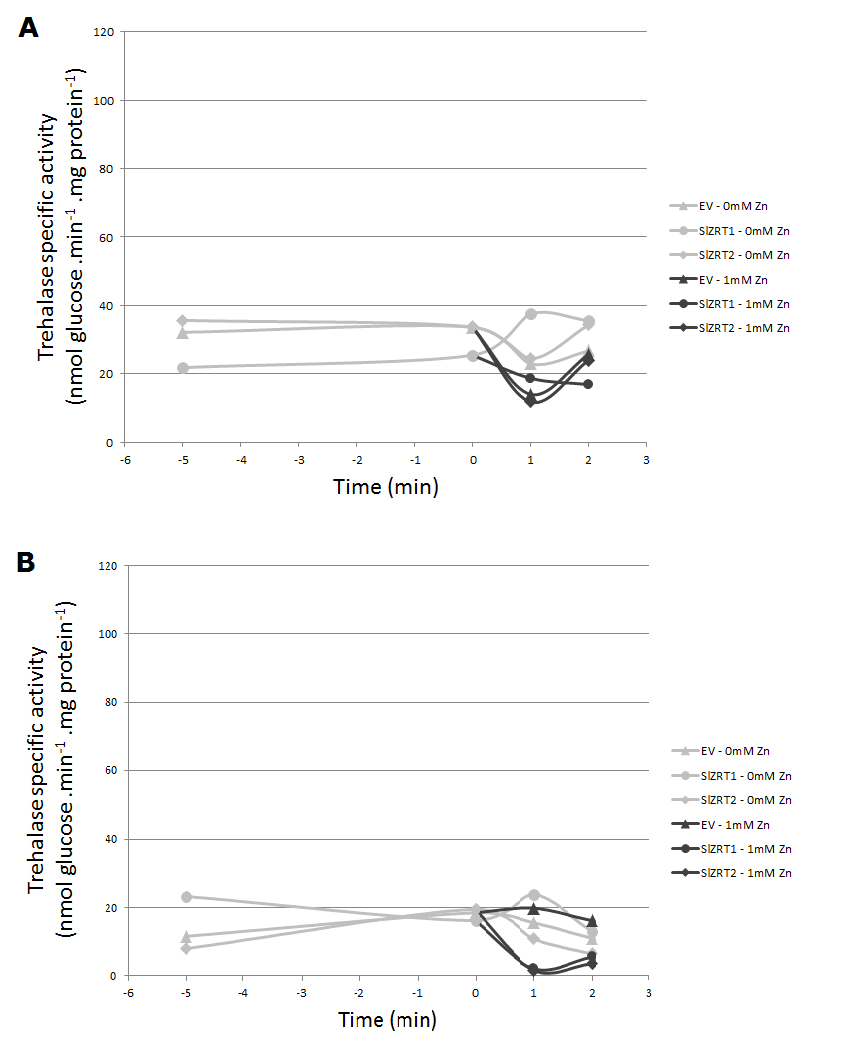

Supplement: FIGURE S5 — Trehalase activity in Zn-depleted Δzrt1Δzrt2 cells after the re-addition of Zn. Δzrt1Δzrt2 cells were transformed with the EV (triangles, pYES-DEST52; Invitrogen), SlZRT1 (circles), or SlZRT2 (diamonds). All yeast cultures were grown for 2 days on SD medium with 0.5 mM citrate to induce Zn starvation. Trehalase activity was assessed in yeast cells maintained on Zn starvation medium (in gray; negative control) and after the addition of 1 mM ZnCl2 (in black) (A) 4.5 h prior to the experiment, yeast cultures were transferred to fresh SD medium with 0.5 mM citrate and 200 μM EDTA. (B) 4 h prior to the experiment, yeast cultures were transferred to fresh SD medium with 10 mM citrate and 1 mM EDTA. [file Image_5.TIF]
